# Mucin-rich environment shapes *Pseudomonas aeruginosa* cytotoxicity and phage-mediated epithelial protection

**DOI:** 10.1016/j.isci.2026.118016

**Published:** 2026-10-28

**Authors:** Daniel O. Patricio, Katri Niitti, Iina Salminen, Lucilene W. Granella, Valtteri Suokko, Gabriel M.F. Almeida, Lotta-Riina Sundberg

**Affiliations:** 1 Department of Biological and Environment Science, Nanoscience Center, University of Jyväskylä, 40014 Jyväskylä, Finland; 2 The Norwegian College of Fishery Science, Faculty of Biosciences, Fisheries and Economics, UiT—The Arctic University of Norway, 9037 Tromsø, Norway

**Keywords:** bacteriophage therapy, mucin-rich environment, *Pseudomonas aeruginosa*, airway epithelial cells, host-pathogen interactions, phage-mediated protection

## Abstract

Chronic respiratory diseases (CRDs) are characterized by excessive mucin accumulation that alters host-pathogen interactions within airway epithelia. Although bacteriophage therapy is increasingly considered as an alternative to antibiotics, it remains unclear how mucin-rich environments influence bacterial virulence and phage efficacy. Here, we show that exogenous mucin reshapes outcomes during *Pseudomonas aeruginosa* in epithelial cells. Mucin exposure induces transcriptional changes, increases epithelial susceptibility to bacterial cytotoxicity, and reduces bacterial adhesion and internalization. Surprisingly, mucin simultaneously enhances phage-mediated protection, preserving epithelial viability without impairing phage amplification. Moreover, prolonged bacterial exposure to mucin increases the cytotoxicity activity of cell-free secretomes, while altering the phenotypic and genotypic characteristics of phage-resistant bacteria. Together, these findings reveal that mucin-rich environments exert opposing effects on infection dynamics by increasing host vulnerability while enhancing phage-mediated control of bacterial infection. Our results highlight the mucosal environment as a key determinant of phage therapy outcomes and bacterial adaptation.

## Highlights

• Mucin increases epithelial susceptibility to *P. aeruginosa* cytotoxicity
• Mucin reduces *P. aeruginosa* internalization in epithelial cells
• Mucin enhances phage-mediated protection of epithelial cells
• Mucin alters phenotypic characteristics of phage-resistant *P. aeruginosa*

## Graphical abstract

**Figure:**
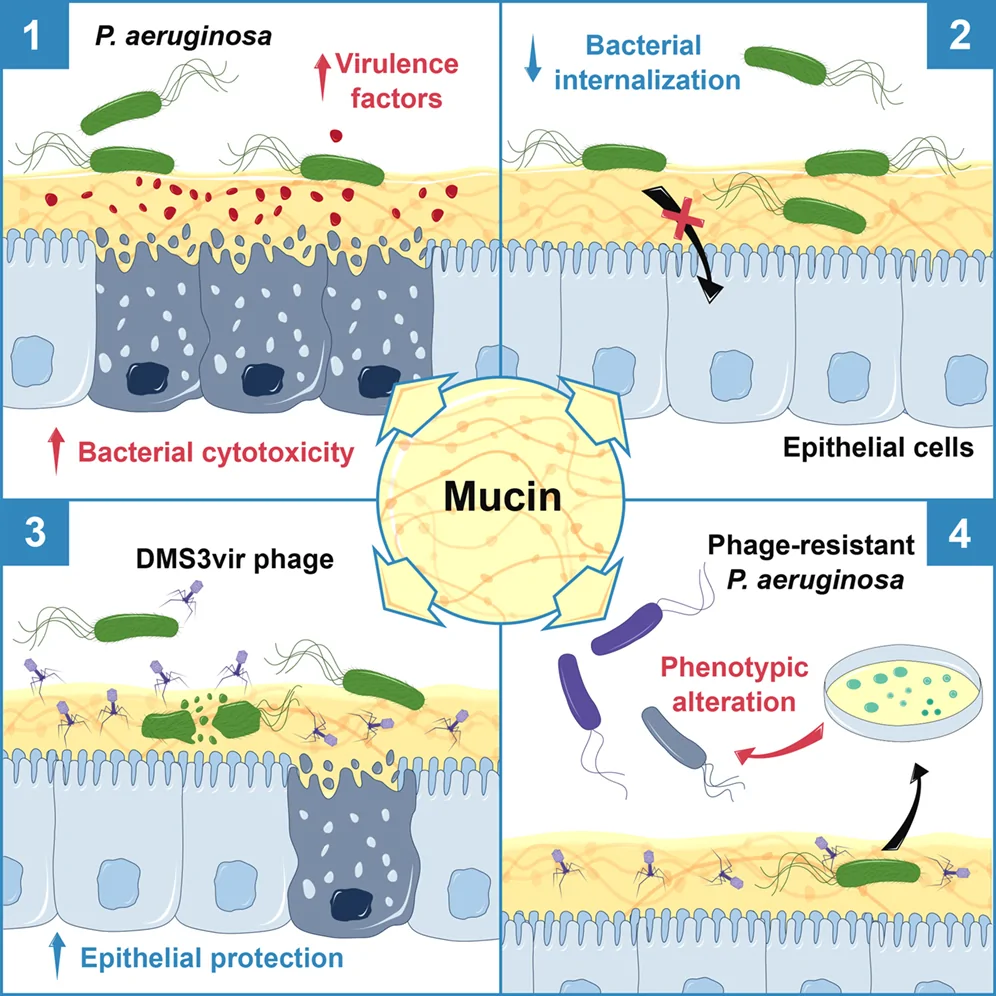

## Teaser

Bacteriology; Biological sciences; Environmental health; Microbiology

## Introduction

Chronic respiratory diseases (CRDs), including cystic fibrosis (CF), chronic obstructive pulmonary disease (COPD), and asthma, are characterized by persistent inflammation, impaired mucociliary clearance, and recurrent airway infections.^1^^,^^2^^,^^3^ A hallmark of these conditions is the hypersecretion of mucins, gel-forming glycoproteins such as MUC5AC and MUC5B, which structure the mucosal layer, trap pathogens, and help maintain epithelial integrity.^1^^,^^2^^,^^3^^,^^4^^,^^5^ Increased mucin production by goblet cells can be influenced by various mediators such as pathogens, inflammatory responses, and xenobiotic exposure.^2^ Mucin overproduction alters physical and biochemical properties of the airway surface, creating a viscous, hypoxic, and oxidatively stressed microenvironment that disrupt immune balance and facilitates microbial colonization.^2^^,^^6^^,^^7^^,^^8^^,^^9^^,^^10^

*Pseudomonas aeruginosa*, a key opportunistic pathogen found in chronic airway infections, thrives under hypoxic conditions. It contributes to epithelial injury and lung function decline through development of antimicrobial resistance, biofilm formation, and secretion of virulence factors regulated by quorum sensing (QS).^11^^,^^12^ The mucosal environment directly shapes *P. aeruginosa* behavior. For instance, mucin glycans have been shown to attenuate virulence by disrupting QS signaling and inhibiting biofilm formation.^13^^,^^14^ Moreover, mucin hyperconcentration contributes to bacterial aggregation that increases antibiotic tolerance variability.^15^^,^^16^ In addition to shaping microbial behavior, mucins are tightly linked to epithelial immune responses, particularly through the regulation of key pro-inflammatory cytokines.^9^^,^^10^

Pro-inflammatory cytokines such as interleukin 6 (IL-6) and IL-8 (or CXCL8) play central roles during CRDs and are associated with mucin hypersecretion.^1^^,^^17^ IL-8 is induced by pathogen-associated molecular patterns (PAMPs) in response to bacterial virulence factors and epithelial stress, acting as a chemoattractant for neutrophils and contributing to sustained inflammation and tissue damage by promoting excessive neutrophil infiltration into the airway lumen.^18^^,^^19^ IL-6, in contrast, is a pleiotropic cytokine involved in acute-phase responses, often upregulated following inflammatory signals, recognition of PAMPs, and secondary pathways.^18^ Elevated levels of IL-6 and IL-8 have been consistently associated with disease severity, frequent exacerbations, and accelerated lung function decline in patients with chronic airway conditions.^20^^,^^21^

The dual effects of mucin highlight a critical gap in our understanding of its influence on the outcome of bacterial infection and treatment. In particular, the effects of mucin-rich environments on modulating bacteriophage (phage), bacterial, and epithelial interactions remain largely undefined.^22^^,^^23^^,^^24^ Phage therapy has re-emerged as a promising approach against multidrug-resistant bacteria due to its specificity, self-amplification, and minimal impact on commensal microbiota.^25^^,^^26^ However, applying phages to mucus-dense tissues presents unique challenges, including limited diffusion, altered receptor accessibility, and potential immune interference.^27^^,^^28^^,^^29^^,^^30^ The “bacteriophage adherence to mucus” (BAM) model proposes that phages bind mucin glycans, increasing their concentration at mucosal surfaces and enhancing pathogen clearance.^31^^,^^32^^,^^33^^,^^34^^,^^35^ Conversely, other studies suggest that mucins may act as a physical barrier, impeding phage diffusion and their ability to reach bacterial hosts.^36^^,^^37^^,^^38^ In other bacterial models, such as *Flavobacterium columnare*, mucins have been shown to trigger bacterial protective stress responses or enhance adaptive mechanisms like CRISPR-Cas immunity.^39^ These contrasting findings underscore the complexity of mucin-phage-bacteria interactions across different biological systems. Thus, the impact of mucins appears highly context-dependent, shaped by mucin type, microbial behavior, and infection dynamics.^29^^,^^39^^,^^40^ In addition, little is known about how mucins influence bacterial evolution under phage pressure. Phage resistance commonly results in fitness costs or trade-offs in host bacteria, including altered motility, surface modifications, or reduced virulence.^41^^,^^42^^,^^43^ Whether mucins mitigate or amplify these trade-offs, and how they shape the phenotypic and genotypic landscape of phage-resistant populations, remains to be determined.

In this study, we used a cell culture model to investigate how a mucin-enriched microenvironment modulates epithelial responses, *P. aeruginosa* cytotoxicity, phage-mediated reduction of epithelial damage, and phenotypic characteristics of phage resistance. We show that mucin exposure induces transcriptional changes consistent with CRD-associated pathways in epithelial cells, enhances bacterial cytotoxicity and inflammation, and selectively represses bacterial virulence pathways. Despite this, mucin enhanced bacteriophage DMS3vir capacity to protect epithelial cells in a bacterial load-dependent manner without eliciting antiviral immune responses. Moreover, mucin shapes the phenotypic and genetic profiles of phage-resistant *P. aeruginosa*, affecting virulent traits and resistance trade-offs. These findings provide new insights into the tripartite interactions between epithelium, bacteria, and phages in mucin-rich conditions with implications for optimizing phage therapies in mucosal environments.

## Results

### Mucin enrichment features CRD-associated transcriptional remodeling in airway epithelial cells

Mucus hypersecretion is a hallmark of chronic airway diseases such as COPD and CF.^2^^,^^4^ To explore how a mucin-rich microenvironment influences epithelial gene expression, we performed RNA sequencing on A549 airway epithelial cells exposed to 0.2% mucin for 18 h. Principal component analysis (PCA) revealed consistent intra-group clustering and clear separation between control and mucin-exposed groups, indicating a transcriptomic shift (Figure 1A). Differential expression analysis identified 179 upregulated and 102 downregulated genes (log_2_ fold change [FC] > 0.5), and a stricter cutoff revealed 38 upregulated and 23 downregulated genes (log_2_FC > 1) in the mucin-exposed cells (Figure 1B). The mucin-rich environment induced regulation of genes associated with cellular stress and inflammation (e.g., response to hypoxia, unfolded protein, and oxidative stress), glucose metabolic shift (e.g., glycolysis and gluconeogenesis), epithelial remodeling (e.g., cell adhesion, junction maintenance, and epithelial-to-mesenchymal transition), and growth-factor signaling (e.g., TGF-β and fibroblast growth factor [FGF]) (Figures 1C and S1).

**Figure 1:**
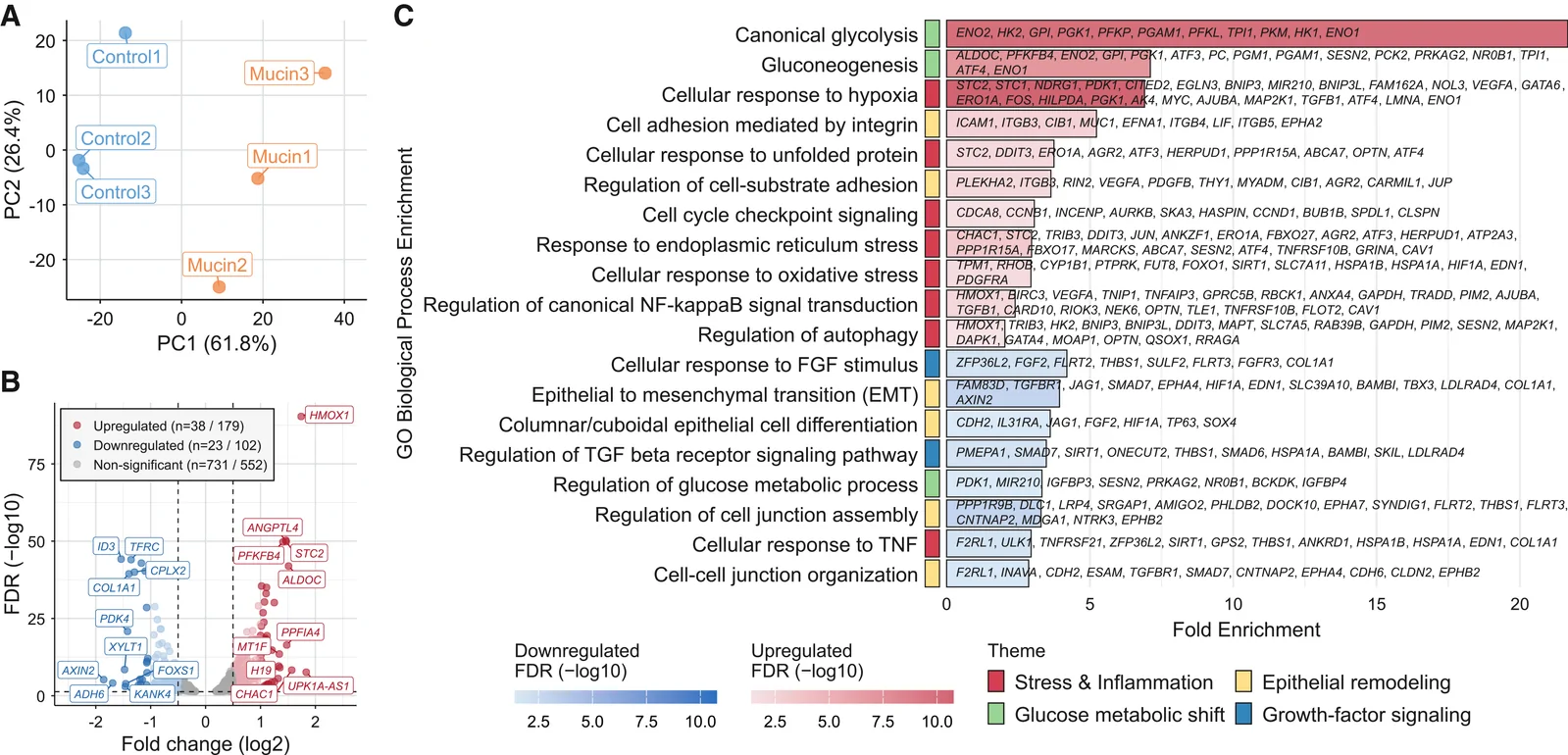
Transcriptomic analysis of A549 cells exposed to a mucin-rich environment (A) Principal component analysis (PCA) of global gene expression in A549 cells incubated with 0.2% mucin or control medium for 18 h (*n* = 3 per group). Each point represents a biological replicate. (B) Volcano plot of differential gene expression between mucin-treated and control cells. Log_2_ fold change (*x* axis) is plotted against the −log_10_ false discovery rate (FDR)-adjusted *p* value (*y* axis). Significantly regulated genes were defined by FDR > 0.5, with log_2_ fold change > 1 or > 0.5 (upregulated, red), and log_2_ fold change > −1 or > −0.5 (downregulated, blue). (C) Gene Ontology (GO) of biological process enrichment analysis of upregulated (red) and downregulated (blue) genes. Bars represent significantly enriched biological processes ranked by fold enrichment, with color intensity corresponding to statistical significance (–log_10_ FDR). See also Figure S1.

These transcriptional changes are consistent with gene expression signatures commonly observed chronic lung disease,^2^^,^^6^^,^^7^ offering a physiologically relevant platform to investigate epithelial responses and epithelial-pathogen interactions under mucosal conditions.

### Mucin-rich environment enhances *P. aeruginosa* cytotoxicity and epithelial inflammatory responses

To investigate how a mucin-rich environment alters bacterial cytotoxicity, we assessed epithelial viability after infecting A549 cells with *P. aeruginosa* strains PA14 and 573. PA14 exhibited significant cytotoxicity at 10^4^ colony-forming units (CFUs), while strain 573 required a higher inoculum (10^6^ CFUs) to elicit similar effects (Figure 2A). Pre-exposure of A549 cells with 0.2% mucin significantly enhanced cytotoxicity induced by both strains at 4 h post-infection, indicating that mucin enrichment sensitizes epithelial cells to bacterial attack (Figure 2B). This effect was independent of exposure timing with mucins, as both pre-incubation and co-incubation with mucin led to similar increases of epithelial cell death (Figure S2A). A similar trend was observed in HT-29 intestinal epithelial cells, where mucin exposure increased susceptibility to PA14-induced cytotoxicity (Figure 2C), suggesting that this phenomenon is not limited to airway epithelial cells. Notably, porcine gastric mucin (PGM) alone had no detectable cytotoxic effect on either cell line, underscoring its role in modulating epithelial-bacteria interactions rather than decreasing cell survival. To explore the underlying mechanisms, we evaluated the impact of mucin on bacterial growth. *P. aeruginosa* PA14 was incubated with 0.2% mucin in DMEM at 37°C for 5 h and quantified by viable colony counting. Bacterial increased CFU counting compared with the input, independently of mucin (Figure 2D), indicating that the increase in bacterial cytotoxicity was not associated with mucin effects on bacterial growth. Next, we investigated whether mucin affects bacterial internalization into epithelial cells. Surprisingly, pre-exposure of A549 cells to 0.2% mucin significantly reduced *P. aeruginosa* uptake in both A549 and HT-29 cells (Figures 2E and 2F). In addition, the mucin effects on bacterial internalization were correlated with a reduction of epithelial cell-associated *P. aeruginosa* PA14 (Figure 2G). Together, these findings indicate that mucin limits bacterial adhesion to the epithelial surface, revealing an inverse relationship between cytotoxicity and bacterial internalization. To determine whether extracellular bacterial products contribute to the increased cytotoxicity, we exposed A549 cells for 5 h to cell-free bacterial secretomes collected after PA14 incubation in the presence of 0.2% mucin (Figure 2H). Secretomes generated in the absence of mucin from either 6 or 24 h were not cytotoxic. Likewise, secretomes from mucin-exposed bacteria collected after 6 h had no detectable effect. In contrast, secretomes obtained after 24 h in the presence of mucin significantly increased epithelial cell death, indicating that mucin promotes the secretion or accumulation of extracellular cytotoxic factors during prolonged bacterial growth. We next assessed whether mucin-rich conditions influence the expression of *IL6* and *IL8*, two key pro-inflammatory cytokines commonly elevated in CRDs.^9^^,^^10^ Both PA14 and 573 strains triggered *IL8* and *IL6* expression after 4 h, and 0.2% mucin further amplified this response under mucin-enriched conditions, while mucin alone did not induce cytokine expression (Figures 2I, 2J, and S2B), indicating that the inflammatory amplification depends on bacterial infection. We observed similar trends in HT-29 cells, where mucin exposure enhanced *IL8* expression during PA14 infection (Figure 2K). However, *IL6* expression remained undetectable in HT-29 cells across all tested conditions (data not shown), consistent with previous findings.^44^^,^^45^

**Figure 2:**
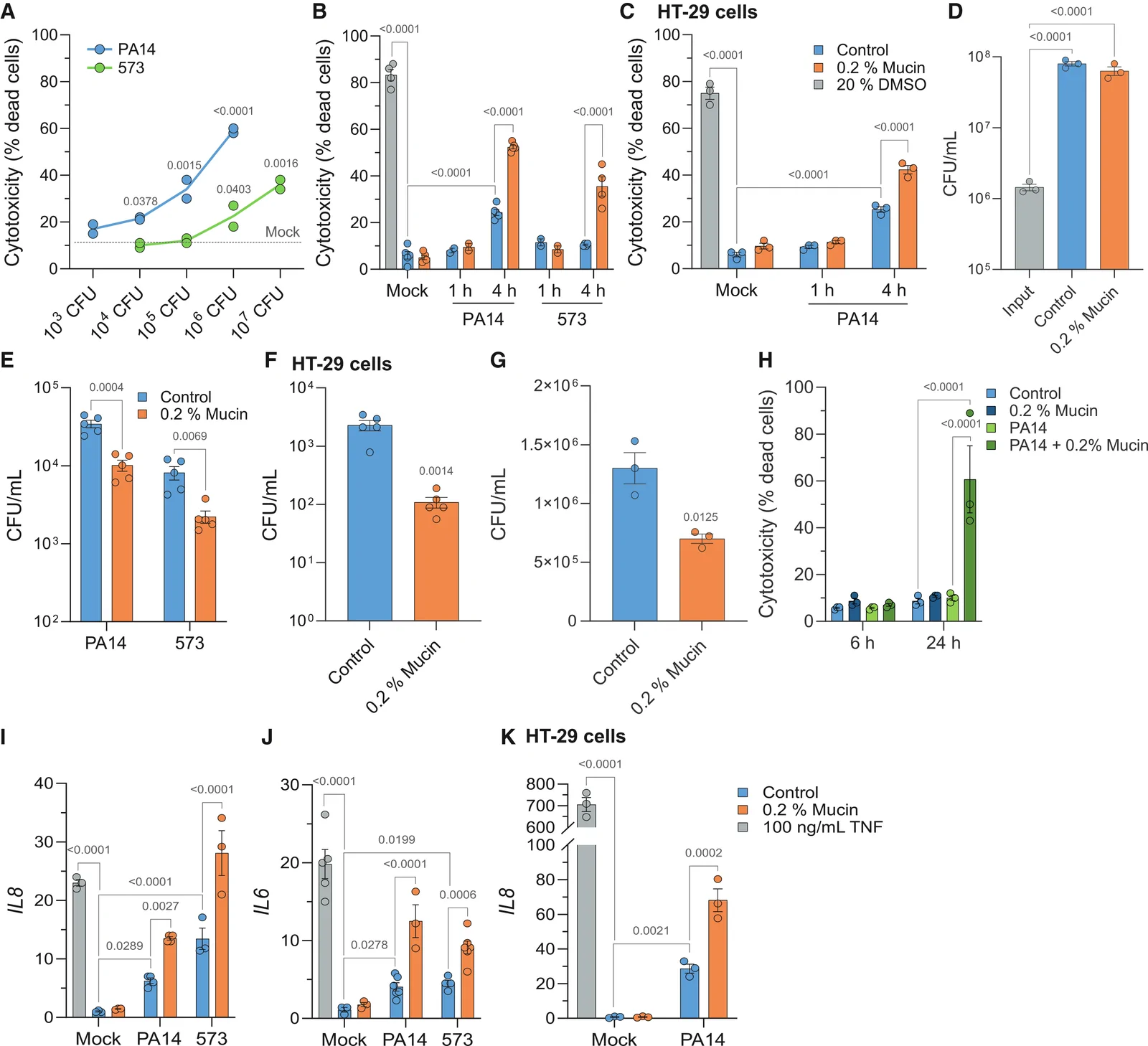
Mucin enrichment increases *P. aeruginosa* cytotoxicity and epithelial inflammatory responses while reduces bacterial internalization (A) Bacterial cytotoxicity on A549 cells infected with *P. aeruginosa* PA14 and 573 strains for 4 h (*n* = 3). (B) Bacterial cytotoxicity on A549 cells pre-incubated with mucin for 18 h prior to infection with PA14 or 573 (1 × 10^5^ CFU). (C) Bacterial cytotoxicity on HT-29 cells pre-incubated with mucin prior PA14 infection (1 × 10^5^ CFU). The 20% DMSO served as a positive control for cytotoxicity. (D) CFU counts of *P. aeruginosa* PA14 exposed to 0.2% mucin in DMEM at 37°C for 5 h under agitation. (E) Bacterial internalization in A549 cells pre-incubated with mucin prior PA14 or 573 infections (1 × 10^5^ CFU) for 1 h. (F) Bacterial internalization in HT-29 cells pre-incubated with 0.2% mucin prior PA14 infection (1 × 10^5^ CFU) for 1 h. (G) Bacterial adhesion on A549 cells pre-exposed with 0.2% mucin prior PA14 infection (1 × 10^5^ CFU) for 1 h. (H) Cytotoxicity of A549 cells exposed for 5 h to cell-free secretomes collected from PA14 (1 × 10^5^ CFU) cultured in DMEM with 0.2% mucin for 6 or 24 h under static condition at 37°C. (I and J) Relative *IL8* (I) and *IL6* (J) mRNA levels in A549 cells pre-exposed with mucin and infected with PA14 or 573 (1 × 10^5^ CFU) for 4 h. (K) *IL8* mRNA expression in HT-29 cells pre-exposed with mucin and infected with PA14 (1 × 10^5^ CFU) for 4 h. The cells were treated with 100 μg/mL TNF as positive control. Quantitative data represent mean ± SEM from indicated biological replicates. Statistical comparisons were performed using two-way ANOVA with multiple comparisons and Sidak’s post hoc test (A–E and H–J) and unpaired two-tailed *t* test (F and G); *p* values and replicate numbers are indicated. Mock, bacterial-free control. See also Figure S2.

Collectively, these results suggest that mucin-rich environments enhance epithelial susceptibility to *P. aeruginosa*, amplifying both cytotoxic and inflammatory responses despite reducing bacterial growth, adhesion, and invasion. Additionally, the extracellular bacterial factors contribute to this phenotype, although direct bacteria-epithelium interactions are likely also required to account for the rapid epithelial damage observed during infection.

### Mucin-enriched environment enhances DMS3vir phage protection of epithelial cells

To evaluate the antibacterial activity of DMS3vir under conditions matching the epithelial infection model, *P. aeruginosa* PA14 was cultured in DMEM with increasing DMS3vir multiplicities of infection (MOIs). As expected, DMS3vir suppressed PA14 growth in a concentration-dependent manner, with higher MOIs producing more sustained bacterial inhibition (Figure 3A). However, bacterial regrowth became evident after approximately 18 h in cultures treated with MOIs of 1 and 10, consistent with the emergence of phage-insensitive bacterial populations during prolonged incubation. Next, we investigated whether mucin interferes with phage amplification. DMS3vir plaque counts increased markedly after 5 h of incubation with PA14 compared with phage-only controls, confirming active phage replication (Figure 3B). Importantly, the presence of 0.2% mucin did not alter phage titers at either MOI tested, indicating that mucin does not impair DMS3vir amplification under the experimental conditions. We next assessed whether mucin affects phage protection of epithelial cells. We co-exposed A549 cells to 0.2% mucin and DMS3vir prior to *P. aeruginosa* PA14 infection. After 4 h, phage pre-incubation reversed mucin-enhanced cytotoxicity, restoring epithelial cell viability to levels similar to non-mucin conditions (Figure 3C). Phage titration confirmed that DMS3vir replicated significantly in the presence of bacteria, and mucin did not alter its amplification (Figure 3D). Extended incubation of 24 h confirmed that phage amplification remained stable regardless of mucin exposure or initial bacterial load (Figures 3E and 3F). Interestingly, 24-h co-culture showed that mucin enhanced phage DMS3vir protective effects of epithelial cells in a manner dependent on the initial bacterial inoculum. At a higher bacteria density (10^5^ CFU), phage alone had limited impact, but the combination with mucin modestly improved epithelial cell viability (Figure 3G). At a lower bacterial inoculum (10^2^–10^3^ CFU), phage and mucin enhanced the preservation of epithelial viability (Figures 3H and 3I), indicating a threshold-dependent enhancement of phage protection of epithelial cells.

**Figure 3:**
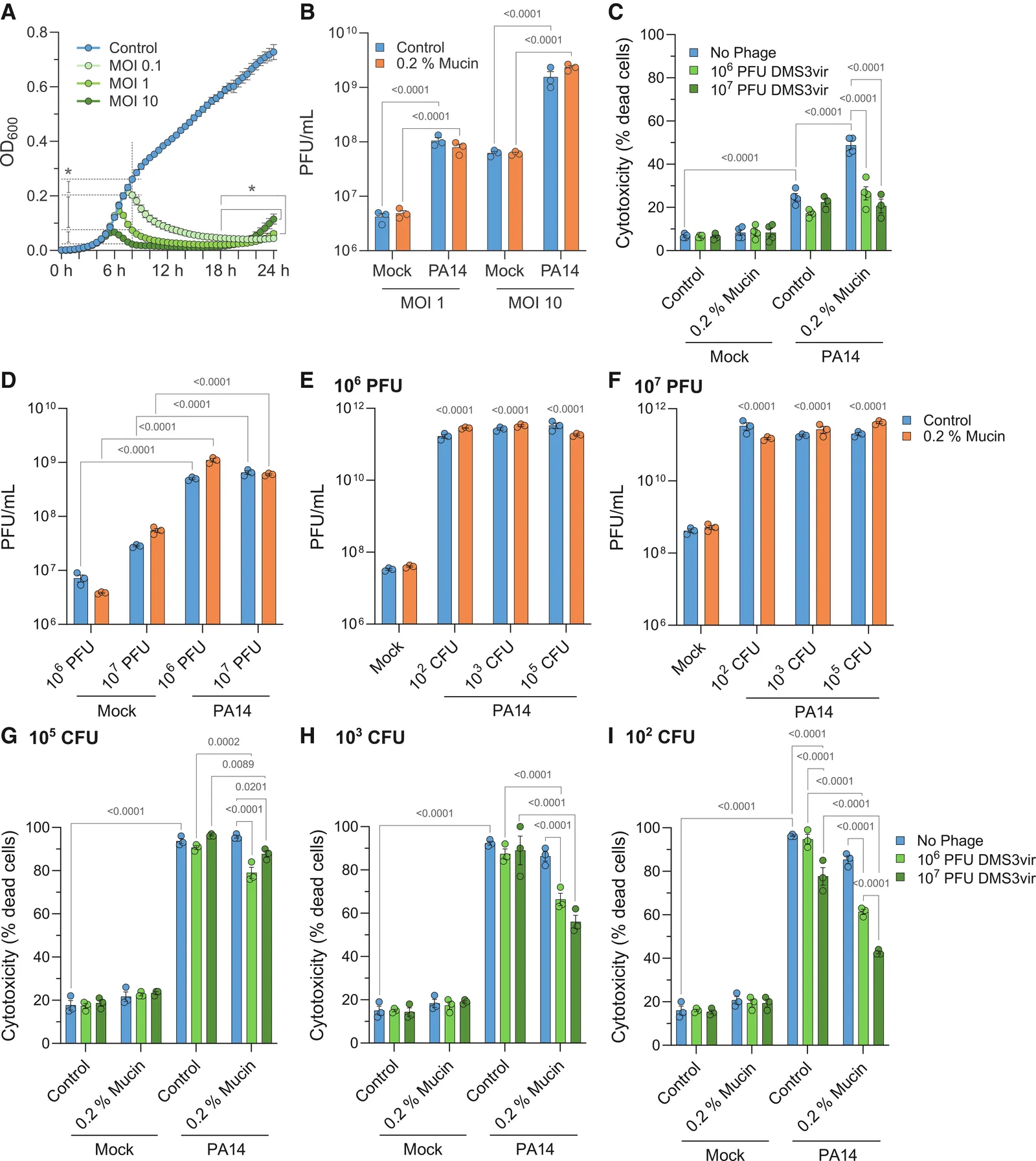
Mucin enrichment enhances the protective activity of phage DMS3vir against *P. aeruginosa* infection (A) Bacterial growth curves of *P. aeruginosa* PA14 (1 × 10^5^ CFU) incubated in DMEM with DMS3vir phage at different multiplicities of infection (MOIs) (*n* = 4). (∗) indicates *p* = <0.0001 between groups at 8 h, or between 18 and 24 h within each group. (B) DMS3vir phage PFU counts following 5 h incubation with PA14 (1 × 10^5^ CFU) in DMEM. Bacterial growth curves of PA14 (1 × 10^5^ CFU) co-exposed with DMS3vir phage (MOI 1) and mucin (*n* = 5). (C) Bacterial cytotoxicity on A549 cells pre-incubated with DMS3vir and mucin for 18 h, followed by infection with PA14 (1 × 10^5^ CFU) for 4 h. (D) DMS3vir quantification of culture supernatants from the same experiment in (C), collected after 4 h of infection. (E and F) Phage quantification from culture supernatants of A549 cells pre-exposed to mucin and DMS3vir 1 × 10^6^ PFU (E) or 1 × 10^7^ PFU prior PA14 infection at varying initial loads (F). (G–I) Bacterial cytotoxicity on A549 pre-exposed to mucin and DMS3vir, following PA14 infection at bacterial initial concentrations of 1 × 10^5^ (G), 1 × 10^3^ (H), or 1 × 10^2^ CFU (I) for 24 h. Quantitative data represent mean ± SEM from the indicated biological replicates. Statistical comparisons were performed using two-way ANOVA with multiple comparisons and Sidak’s post hoc test (A–I); *p* values and replicate numbers are indicated. Mock, bacterial-free control.

We then examined whether phage treatment influences epithelial inflammatory responses under mucin-rich conditions. As previously shown, mucin amplified *IL6* and *IL8* expression during PA14 infection (Figures 4A and 4B). Interestingly, DMS3vir incubation reduced *IL8* expression regardless of mucin, while *IL6* remained unaffected. In addition, phage exposure did not trigger antiviral signaling pathways, as *IFNB* levels remained stable (Figure S2C) and *IFNL1* transcripts were undetectable under the experimental conditions tested.

**Figure 4:**
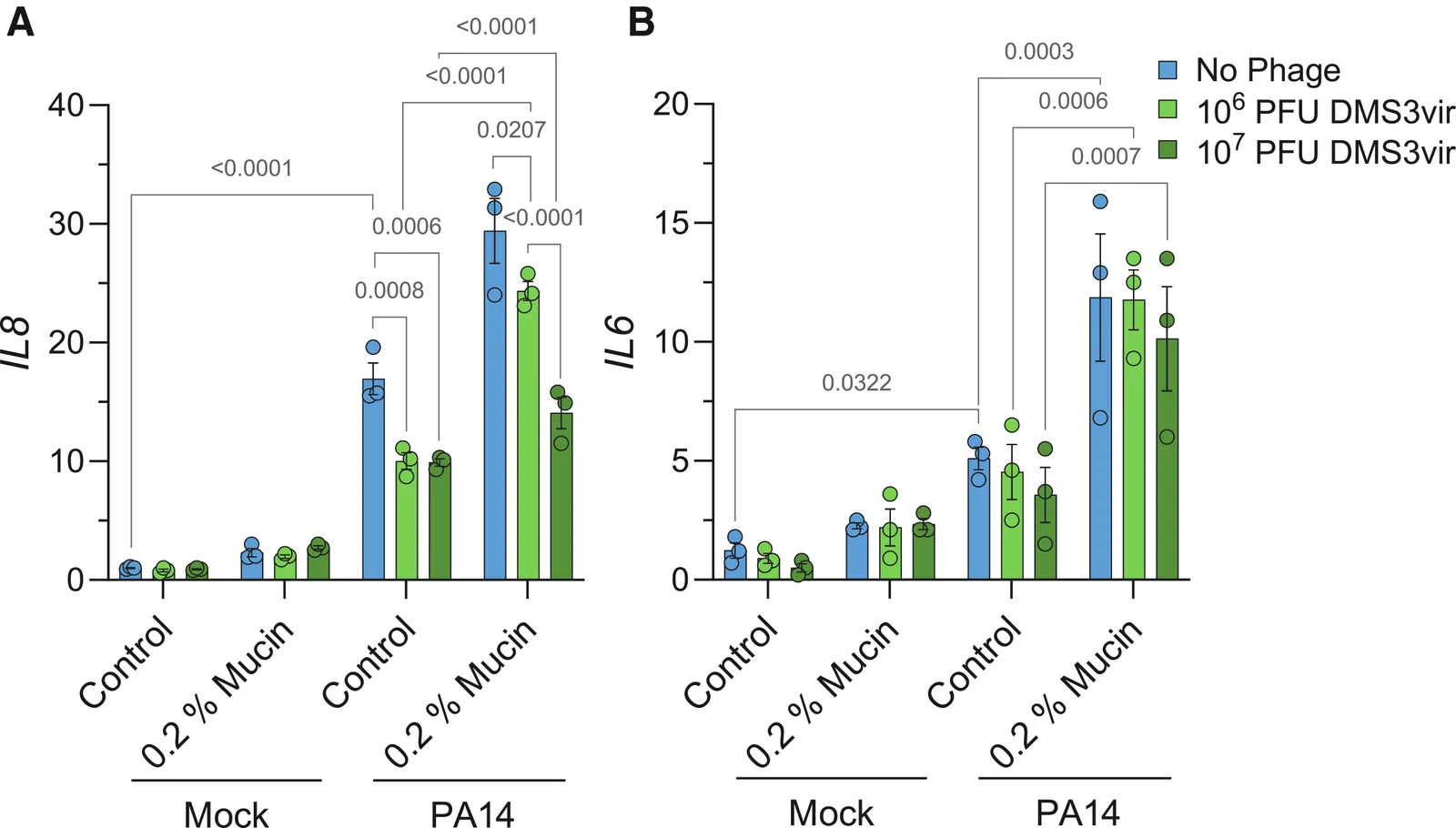
Phage DMS3vir attenuates *IL8*, but not *IL6*, expression in *P. aeruginosa*-infected epithelial cells (A) Relative expressions of *IL8* and (B) *IL6* mRNA of A549 cells pre-incubated with DMS3vir and mucin for 18 h, followed by infection with *P. aeruginosa* PA14 (1 × 10^5^ CFU) for 4 h. Quantitative data represent mean ± SEM from the indicated biological replicates. Statistical comparisons were performed using two-way ANOVA with multiple comparisons and Sidak’s post hoc test (A and B); *p* values and replicate numbers are indicated. Mock, bacterial-free control.

These results demonstrate that mucin-rich microenvironments enhance the epithelial protective efficacy of DMS3vir phage without impairing its replication, especially at physiologically relevant bacterial densities. Moreover, DMS3vir exerts selective immunomodulatory effects while avoiding antiviral responses, highlighting its compatibility with mucosal tissues.

### Mucin-enriched environment modulates phenotypic traits in phage-resistant *P. aeruginosa*

To investigate whether a mucin-enriched environment affects the development of phage resistance, we co-incubated A549 cells with 0.2% mucin and DMS3vir phage for 18 h prior to *P. aeruginosa* PA14 infection. After 24 h of bacterial exposure, we isolated six independent bacteriophage-insensitive mutants (BIMs): three from standard conditions (BIM1–3), three from mucin-enriched conditions (BIM(M)1–3), and three control isolates (not exposed to phage) including PA14(WT) (ancestral strain), PA14(C) (A549-exposed), and PA14(M) (A549 + mucin). The control isolated colonies retained their characteristic smooth, mucoid, and yellow-green colony morphology (Figures 5A and S3A). In contrast, BIM clones developed small, rough colonies with enhanced green pigmentation, suggesting increased pyocyanin production (Figures 5B and S3A). BIM(M) isolates displayed intermediate morphologies, transitioning between rough and smooth colonies with yellowish-green pigmentation, although the BIM(M)1 isolate retained a rough (Figures 5C and S3A). Isolates exposed to DMS3vir phage confirmed bacterial resistance of BIM and BIM(M) clones (Figures S3B–S3D). Whole-genome sequencing revealed mutations in genes associated with virulence, motility, attachment, and metabolism, while CRISPR analysis showed no new spacer acquisition, indicating resistance likely resulted from surface modification (Figures S4A–S4C).

**Figure 5:**
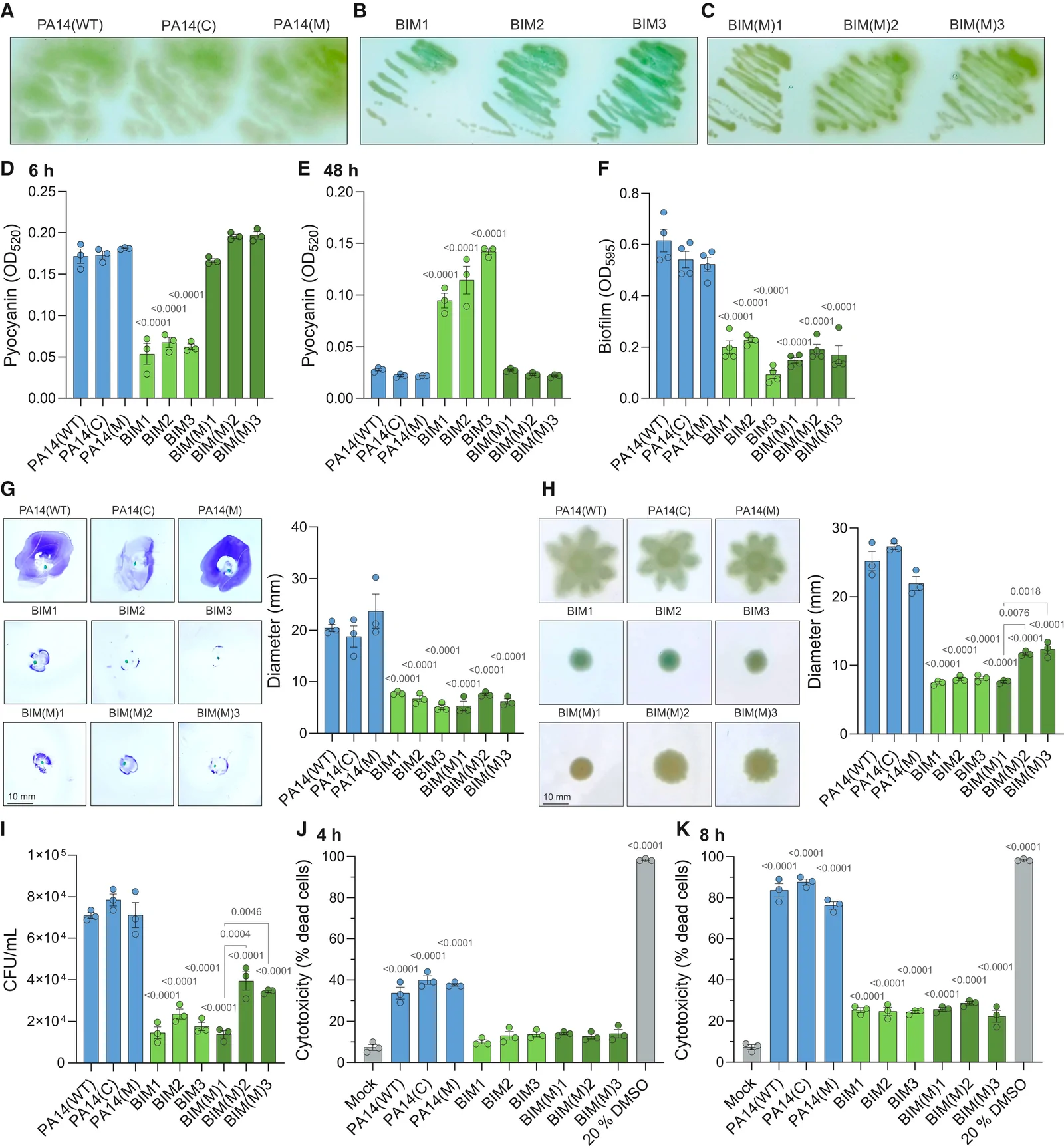
Phenotypic characterization of phage-resistant *P. aeruginosa* mutants reveals mucin-dependent modulation of virulence traits (A–C) Representative colony images of *P. aeruginosa* PA14 clones isolated after 24 h co-culture at 1 × 10^5^ CFU with A549 cells pre-exposed to 0.2% mucin and DMS3vir phage (1 × 10^7^ PFU). (D and E) Pyocyanin production by PA14 clones (1 × 10^8^ CFU) after 6 h (D) or 48 h (E) incubation in LB medium. (F) Biofilm formation measured by crystal violet staining (OD_595_) after 24 h at 37°C under static conditions. (G) Twitching motility: representative images (left) and motility diameters (right) of PA14 clones after 48 h at 37°C, stained with crystal violet. (H) Swarming motility: representative images (left) and motility diameters (right) of PA14 clones spotted (2 μL, OD_600_ = 0.1) after 24 h at 37°C. (I) Bacterial internalization (2 × 10^5^ CFU) in A549 cells for 1 h. (J and K) Cytotoxicity in A549 cells infected with PA14 clones (1 × 10^5^ CFU) for 4 h (J) or 8 h (K). The 20% DMSO treatment served as a positive control for cytotoxicity. Quantitative data represent mean ± SEM from the indicated biological replicates. Statistical comparisons were performed using one-way ANOVA with Tukey’s post hoc test (D–H); *p* values and replicate numbers are indicated. PA14(WT), ancestral strain; PA14(C), isolated from PA14 + A549; PA14(M), isolated from PA14 + A549 + mucin; BIM1–3, bacteriophage-insensitive mutants (BIMs) isolated from PA14 + A549 + DMS3vir; BIM(M)1–3, BIMs isolated from PA14 + A549 + DMS3vir + mucin; Mock, bacterial-free control. See also Figures S3 and S4.

Phenotypic assays revealed distinct traits associated with phage resistance. After 6 h, BIM(M) strains produced pyocyanin at levels comparable to the control group, whereas BIM strains showed reduced production (Figure 5D). At 48 h, BIM strains increased pyocyanin levels, in contrast with reduced production in BIM(M) and control clones (Figure 5E). Despite these differences, both phage-resistant groups formed weaker biofilms than controls (Figure 5F), indicating that impaired biofilm formation represents a shared cost of resistance. Motility assays further distinguished the groups. All BIM and BIM(M) isolates lost twitching, a type IV pili-dependent motility (Figure 5G). Moreover, BIM(M)2 and BIM(M)3 retained partial swarming motility (flagella-dependent), whereas BIM strains and BIM(M)1 were immotile (Figure 5H). This swarming behavior correlated with bacterial internalization capacity, where all BIM strains and BIM(M)1 showed reduced entry into epithelial cells, while BIM(M)2 and BIM(M)3 retained partial entry compared to controls (Figure 5I). Moreover, cell viability assays confirmed reduced cytotoxicity across all phage-resistant clones. Both BIM and BIM(M) strains induced minimal epithelial cell death at 4 h and 8 h post-infection compared to controls (Figures 5J and 5K).

Together, these results demonstrate that mucin-rich microenvironments do not prevent the emergence of phage resistance but influence the phenotypic and genotypic landscape of resistant populations. BIM(M) strains retained pyocyanin levels and partially preserved swarming motility and internalization, while maintaining reduced cytotoxicity, impaired biofilm formation, and loss of twitching motility. These findings underscore the complex interplay between mucosal environments, bacterial adaptation, and phage-mediated selection, and highlight the importance of epithelial-derived factors in shaping the evolutionary outcomes relevant to phage therapy.

## Discussion

CRDs, including COPD and CF, are characterized by excessive mucin accumulation that profoundly alters the airway microenvironment and host-pathogen interactions.^7^^,^^9^^,^^46^ In this study, we show that a mucin-enriched environment reshapes the outcomes during *P. aeruginosa* infection by exerting opposing effects on epithelial susceptibility and phage-mediated control. Specifically, mucin exposure induces transcriptional changes in epithelial cells consistent with inflammatory and stress-associated pathways, increases epithelial susceptibility to *P. aeruginosa* despite reducing bacterial adhesion and internalization, promotes the accumulation of extracellular bacterial cytotoxic factors after prolonged incubation, and enhances the protective effects of bacteriophage DMS3vir without impairing phage amplification. In addition, mucin influences the phenotypic characteristics of phage-resistant isolates recovered after phage exposure. Together, these findings identify mucin as a key environmental determinant of bacterial pathogenesis and phage therapy efficacy.

First, we aimed to understand how exogenous mucins influence epithelial cells. Our transcriptomic analysis indicates that exposure to exogenous mucin is sufficient to induce gene expression patterns in epithelial cells that are consistent with those observed in CRDs.^3^^,^^5^^,^^6^^,^^7^ These include pathways related to hypoxia, oxidative stress, metabolic reprogramming, and epithelial remodeling. Such changes suggest that mucin-rich environments may not only serve as a physical barrier but also actively modulate epithelial physiology. Rather than acting as a passive component of mucus, mucin may contribute to shaping epithelial responses in ways that alter responses during subsequent bacterial infection.

Next, we explored the effects of mucin on bacterial and epithelial cells interaction. Surprisingly, mucin exposure increases epithelial susceptibility to *P. aeruginosa* while simultaneously reducing bacterial adhesion and internalization. This phenotype cannot be explained by enhanced bacterial invasion or increased early bacterial proliferation, as mucin does not alter *P. aeruginosa* growth during early incubation period used for infection assays. Instead, exposure of epithelial cells to bacterial-free secretomes, generated from prolonged bacterial growth in the presence of mucin, enhances extracellular factors that induce substantial epithelial cytotoxicity. Together, these findings suggest that enhanced epithelial damage arises primarily from altered extracellular bacterial activity rather than increased bacterial entry into host cells. One possible explanation is that soluble mucin competes with membrane-associated mucins such as MUC1 for bacterial binding. MUC1 interacts with *P. aeruginosa* flagellin and facilitates bacterial adhesion to epithelial surfaces.^47^^,^^48^ Excess extracellular mucin may therefore reduce bacterial attachment by masking epithelial binding sites while prolonging bacterial residence within a mucin-rich environment that favors the accumulation or activity of extracellular virulence factors. Although the specific extracellular factors responsible remain unidentified, our findings demonstrate that secreted bacterial products contribute to epithelial injury. Because secretomes generated after 6 h were not cytotoxic whereas direct bacterial infection induced epithelial damage within the same period, direct bacteria-epithelium interactions are also likely to participate in the early stages of infection. In addition, mucin-mediated alterations in bacterial behavior, including changes in flagellar motility and aggregation, may further influence bacterial access to epithelial cells, as previously linked to aggregation-mediated tolerance in mucin environments.^16^ Alternatively, mucin-induced epithelial stress responses may sensitize host cells to bacterial products, amplifying cytotoxic outcomes without requiring increased bacterial entry. These interpretations are consistent with the observed increase in pro-inflammatory cytokine expression, particularly IL-6 and IL-8, commonly found during mucosal infections.^17^^,^^20^^,^^49^ Increase in IL-8 may amplify neutrophil recruitment and contribute to tissue damage,^50^ reinforcing a detrimental cycle of inflammation and epithelial remodeling. Together, these findings support a model in which mucin-rich environments enhance epithelial responsiveness to bacterial factors rather than promoting intracellular infection.

Strikingly, mucin also enhanced the ability of phage treatment to reduce epithelial cytotoxicity. Although mucin did not affect DMS3vir amplification or lytic activity in bacterial cultures, its presence improved epithelial protection against bacterial infections, particularly at lower bacterial densities. One potential explanation is that mucin alters the spatial organization of bacteria and phages, increasing the likelihood of productive encounters. This interpretation is consistent with the BAM model, in which phages bind mucin glycans and accumulate at mucosal surfaces, thereby enhancing their capacity to control bacterial populations.^31^^,^^32^^,^^33^^,^^34^^,^^35^ Although we did not directly measure phage-mucin interactions, the enhanced protective effect observed under mucin-rich conditions supports the idea that mucus can facilitate phage activity in complex biological environments. In this context, mucosal environments could potentially be leveraged to enhance phage-based interventions, as supported by recent inhaled phage studies in patients with CF reporting reduced *P. aeruginosa* burden without adverse events.^51^

Phage DMS3vir exposure further modulated epithelial immune responses during *P. aeruginosa* infection by reducing *IL8* expression without affecting *IL6* or inducing antiviral signaling pathways. Because DMS3vir alone or supplemented with mucin did not alter cytokine expression, the preferential reduction of *IL8* likely reflects decreased bacterial stimulation following phage-mediated bacterial lysis rather than selective targeting of epithelial signaling pathways.^19^^,^^52^ IL-8 is a major neutrophil chemoattractant, and its excessive production contributes to persistent neutrophilic inflammation and progressive tissue damage in chronic airway diseases.^17^ Thus, reducing *IL8* expression may represent an additional therapeutic benefit of phage treatment by limiting excessive inflammatory responses while preserving antibacterial activity. In contrast, IL-6 is regulated by a broader range of inflammatory and stress-associated pathways and may therefore be less sensitive to the reduction in viable bacteria during the early stages of infection.^19^^,^^53^ Furthermore, the absence of detectable type I or III interferon responses indicates that DMS3vir phage does not trigger a measurable antiviral response in epithelial cells,^54^ supporting the compatibility of phage therapy with mucosal tissues.

Our results also demonstrate that mucin exposure influences the phenotypic characteristics of phage-resistant *P. aeruginosa*. Although resistance emerged in both conditions, mucin-exposed populations displayed distinct phenotypic and genotypic profiles, including differences in pigmentation, motility, and internalization capacity, underscoring the role of mucin in shaping resistance trajectories.^31^^,^^32^^,^^39^^,^^55^^,^^56^ The absence of CRISPR spacer acquisition supports phage resistance by surface modification as the dominant mechanism.^57^^,^^58^^,^^59^ Loss of type IV pili-mediated twitching motility was observed uniformly across all phage-resistant clones, indicating that pili function represents a common and high-cost target during resistance evolution. This phenotype is consistent with the role of type IV pili as the primary receptor for DMS3vir phage,^60^ suggesting that resistance arises through receptor modification. However, the fitness cost of pili disruption extends beyond phage resistance, as twitching motility is required for early surface colonization, microcolony expansion, and biofilm initiation.^15^ In contrast, flagella-dependent swarming motility was completely abolished in BIM strains and in BIM(M)1, but only partially impaired in BIM(M)2–3 isolates. This divergence suggests that mucin-rich environments may buffer some of the pleiotropic costs associated with resistance and preserve flagellar function under selective pressure. Because *P. aeruginosa* flagellin interacts with MUC1 on airway epithelial cells,^47^^,^^48^ the retention of flagella has direct implications for bacterial adhesion and cellular uptake. Accordingly, partial swarming in BIM(M) isolates corresponds to a moderate reduction in bacterial internalization, whereas the complete loss of swarming in BIM and BIM(M)1 is associated with a strong reduction in epithelial entry. Notably, while all resistant clones exhibited reduced cytotoxicity and impaired biofilm formation, mucin-exposed isolates retained selected virulence-associated traits. Delayed pyocyanin accumulation in BIM strains may reflect altered QS or metabolic adaptation,^61^^,^^62^^,^^63^ supporting the idea that mucin can modulate resistance-associated fitness costs and enable the retention of specific virulence phenotypes. Although this analysis was based on a limited number of phage-resistant isolates, these findings suggest that mucin-rich environments impose additional selective pressures that shape the range of viable adaptive strategies, highlighting the importance of environmental context in modulating phenotypic variations of phage-exposure pathogens.

Several mechanisms may underlie the dual effects of mucin observed in this study. Our adhesion and internalization assays indicate that soluble mucin reduces bacterial association with epithelial cells, suggesting that mucin alters the epithelial interface rather than promoting bacterial invasion. In contrast, prolonged bacterial exposure to mucin generated cell-free secretomes with increased cytotoxic activity, indicating that extracellular factors contribute to epithelial damage. In addition, mucin did not impair DMS3vir amplification, and phage treatment consistently protected epithelial cells from bacterial cytotoxicity. These findings are consistent with the BAM model, in which mucosal environments may facilitate interactions between phages and bacteria, although phage retention, diffusion, and adsorption to mucin were not directly investigated.

In summary, mucin-rich environments increased epithelial susceptibility to *P. aeruginosa* while enhancing phage-mediated epithelial protection. By reshaping epithelial responses, bacterial adhesion, extracellular cytotoxic activity, phage performance, and evolutionary trajectories, mucin emerges as an important determinant of infection outcome. These findings underscore the importance of considering the mucosal environment when developing phage-based therapeutic strategies.

### Limitations of the study

While this study provides new mechanistic insights into the interplay between mucin, bacteria, phage, and epithelial cells, several limitations should be acknowledged. A549 and HT-29 cells are cancer-derived models and do not fully reproduce the complexity of the airway epithelium. Although we included both the highly virulent *P. aeruginosa* laboratory strain PA14 and the less virulent clinical isolate 573, most mechanistic experiments were performed with PA14.^32^^,^^64^^,^^65^ Therefore, the findings should be interpreted cautiously, as they may not fully represent the diversity of *P. aeruginosa* isolates associated with chronic respiratory infections. In addition, PGM, although widely used as an experimental model due to its abundant human-like MUC5AC and glycosylated structure, does not fully reflect the biochemical and biophysical complexity of human airway mucus, which contains multiple mucins, antimicrobial proteins, and other host-derived components.^39^^,^^66^^,^^67^^,^^68^ These compositional differences may influence bacterial sensing, virulence regulation, and phage behavior. The concentration of 0.2% PGM was selected because it is widely used in mucin-bacteria studies and, in our model, induced epithelial transcriptional remodeling and bacteria-dependent responses without affecting epithelial viability or substantially altering bacterial growth. Different mucin concentrations may differentially influence the phenotype output. Finally, although our adhesion and secretome experiments provide mechanistic insight into the increased epithelial susceptibility, the specific bacterial factors involved and the contribution of the BAM model remain to be determined. Future studies using differentiated airway models, clinical isolates, diverse mucin sources, and multiple bacteriophages will be important to validate and extend these findings.

## Resource availability

### Lead contact

Requests for further information and resources should be directed to and will be fulfilled by the lead contact, Lotta-Riina Sundberg (lotta-riina.sundberg@jyu.fi).

### Materials availability

All unique/stable reagents generated in this study are available from the lead contact with a completed materials transfer agreement.

### Data and code availability

• Processed RNA-seq data have been deposited at GEO, and the corresponding raw sequencing reads are available through NCBI SRA. Raw bacterial whole-genome sequencing reads are also available through NCBI SRA. Additional datasets generated in this study have been deposited at Mendeley Data. All datasets are publicly available as of the date of publication, and accession numbers and DOI information are listed in the key resources table. Data reported in this paper will be shared by the lead contact upon request.
• The original code used in this study has been deposited at Mendeley Data and is publicly available as of the date of publication. The DOI is listed in the key resources table.
• Any additional information required to reanalyze the data reported in this paper is available from the lead contact upon request.

## Acknowledgments

We would like to thank Jenne Auramo and Petri Papponen for their help in the laboratory, Dr. Nina Chanisvili (George Eliava Institute of Bacteriophages, Microbiology & Virology, Tbilisi, Georgia) for donating *P. aeruginosa* strain 573, Prof. Ville Friman (University of Helsinki) for donating the phage DMS3vir, and Prof. Varpu Marjomäki (University of Jyväskylä) for providing the enterovirus *Coxsackievirus A9* (CVA9) used as a positive control for *IFNB* expression. The research was funded by 10.13039/501100002341Research Council of Finland grant #346772 (L.-R.S.) and the Centre for New Antibacterial Strategies (CANS) of the Arctic University of Norway #2520855 (G.M.F.A.).

## Author contributions

Conceptualization, D.O.P., G.M.F.A., and L.-R.S.; data curation, D.O.P.; formal analysis, D.O.P.; funding acquisition, L.-R.S.; investigation, D.O.P., K.N., I.S., L.W.G., and V.S.; methodology, D.O.P., V.S., G.M.F.A., and L.-R.S.; project administration, D.O.P. and L.-R.S.; resources, L.-R.S.; software, D.O.P.; supervision, G.M.F.A. and L.-R.S.; validation, D.O.P., G.M.F.A., and L.-R.S.; visualization, D.O.P.; writing – original draft, D.O.P.; writing – review and editing, D.O.P., K.N., I.S., L.W.G., V.S., G.M.F.A., and L.-R.S.

## Declaration of interests

We declare that L.-R.S. and G.M.F.A. are owners of a patent titled “Improved methods and culture media for production, quantification, and isolation of bacteriophages” (https://patentscope.wipo.int/search/en/WO2019150003).

## STAR★Methods

### Key resources table

**Table:** 

REAGENT or RESOURCE	SOURCE	IDENTIFIER
**Bacterial and virus strains**		

*P. aeruginosa* strain PA14	Laboratory stock	DSM 19882
*P. aeruginosa* strain 573	Laboratory stock	Freschi et al.^64^
Bacteriophage DMS3vir	Laboratory stock	Cady et al.^69^
Phage-resistant *P. aeruginosa* clones	This paper	N/A

**Chemicals, peptides, and recombinant proteins**		

Dulbecco’s Modified Eagle Medium (DMEM)	Thermo Fisher Scientific	Cat# 52100039
Fetal Bovine Serum (FBS)	Thermo Fisher Scientific	Cat# A5256701
GlutaMAX™ Supplement	Thermo Fisher Scientific	Cat# 35050038
Penicillin-Streptomycin	Thermo Fisher Scientific	Cat# 15140122
Type III porcine gastric mucin	Sigma-Aldrich	Cat# M1778
Human TNF-alpha Recombinant Protein	Thermo Fisher Scientific	Cat# PHC3011
Dimethyl sulfoxide (DMSO)	Sigma-Aldrich	Cat# D8418
Trypsin-EDTA	Thermo Fisher Scientific	Cat# 15400054
Trypan Blue Solution	Thermo Fisher Scientific	Cat# 15250061
Gentamicin	Thermo Fisher Scientific	Cat# 15710064
Triton™ X-100	Sigma-Aldrich	Cat# X100
Chloroform	Sigma-Aldrich	Cat# C2432
Crystal violet	Sigma-Aldrich	Cat# C0775

**Critical commercial assays**		

RNeasy Mini Kit	Qiagen	Cat# 74106
High-Capacity cDNA Reverse Transcription Kit	Thermo Fisher Scientific	Cat# 4368813
GoTaq® qPCR Master Mix	Promega	Cat# A6002
EndoTrap® HD 1/1	Lionex	Cat# LET0009
Pierce™ Chromogenic Endotoxin Quant Kit	Thermo Fisher Scientific	Cat# A39552S
DNeasy Blood & Tissue Kit	Qiagen	Cat# 69504

**Deposited data**		

RNA-seq data	GEO	GSE307737
Raw RNA-seq and Whole-genome sequencing data	NCBI SRA (BioProject)	PRJNA1308451
Datasets and original code	Mendeley Data	https://doi.org/10.17632/z4rkkpyxcj.1

**Experimental models: Cell lines**		

A549	ATCC	RRID:CVCL_0023
HT-29	ATCC	RRID:CVCL_0320

**Oligonucleotides**		

Myc_526_Forward: ACATTGGGACTGAGATACGGC	This paper	N/A
Myc_1031_Reverse: TCGTTTACGGCGTGGACTAC	This paper	N/A
18S_Forward: TAGAGGGACAAGTGGCGTTC	This paper	N/A
18S_Reverse: CGCTGAGCCAGTCAGTGT	This paper	N/A
hIL6_Forward: CCACACAGACAGCCACTCAC	This paper	N/A
hIL6_Reverse: AGGTTGTTTTCTGCCAGTGC	This paper	N/A
hIL8_Forward: ACTGAGAGTGATTGAGAGTGGAC	This paper	N/A
hIL8_Reverse: AACCCTCTGCACCCAGTTTTC	This paper	N/A
hIFNB_Forward: AAACTCATGAGCAGTCTGCA	This paper	N/A
hIFNB_Reverse: AGGAGATCTTCAGTTTCGGAGG	This paper	N/A
hIFNL1_Forward: TTCCAAGCCCACCACAACTG	This paper	N/A
hIFNL1_Reverse: GAGTGACTCTTCCAAGGCGT	This paper	N/A
oprL_Forward: AACAGCGGTGCCGTTGAC	This paper	N/A
oprL_Reverse: GTCGGAGCTGTCGTACTCGAA	This paper	N/A
algD_Forward: GCGACCTGGACCTGGGCT	This paper	N/A
algD_Reverse: GCGACCTGGACCTGGGCT	This paper	N/A
rhlR_Forward: GCGTCAAGAAAGAAACCCGTCTG	This paper	N/A
rhlR_Reverse: GGCGTAAATGTGCTGGGAAGAG	This paper	N/A
rhlI_Forward: CTCTCTGAATCGCTGGAAGG	This paper	N/A
rhlI_Reverse: CGACGATGTAGCGGGTTT	This paper	N/A
lasR_Forward: GCGCCATCGGCAAGACCAG	This paper	N/A
lasR_Reverse: GCGGGAGGTCACACCGAAC	This paper	N/A
lasI_Forward: GTAGGCGTGGAGAAGATGATG	This paper	N/A
lasI_Reverse: ATCTGGGTCTTGGCATTGAG	This paper	N/A

**Recombinant DNA**		

p_m16S plasmid	Addgene	Cat# 160549

**Software and algorithms**		

GraphPad Prism (v10.3.0)	GraphPad	RRID:SCR_002798
ImageJ2 (v2)	Schneider et al.^70^	RRID:SCR_003070
R (v4.5.1)	R Foundation	RRID:SCR_001905
FastQC (v0.12.1)	Babraham Institute	RRID:SCR_014583
MultiQC (v1.31)	Ewels et al.^71^	RRID:SCR_014982
fastp (v0.23.4)	Chen et al.^72^	RRID:SCR_016962
STAR (v2.7.11b)	Dobin et al.^73^	RRID:SCR_015899
HTSeq (v2.0.3)	Anders et al.	RRID:SCR_005514
BWA-MEM (v0.7.19)	Li et al.^74^	RRID:SCR_010910
SAMtools (v1.21)	SAMtools project	RRID:SCR_002105
GATK toolkit (v4.6.2)	DePristo et al.^75^	RRID:SCR_001876
VarScan (v2.4.2)	Koboldt et al.^76^	RRID:SCR_006849
bcftools (v1.12)	https://doi.org/10.1101/090811	RRID:SCR_005227
SPAdes (v4.0.0)	Nurk et al.^77^	RRID:SCR_000131
MinCED (v0.2.0)	Bland et al.^78^	N/A
DESeq2 (v1.50.0)	Bioconductor	RRID:SCR_015687
edgeR (v4.18.1)	Bioconductor	RRID:SCR_012802
VariantAnnotation (v1.52.0)	Bioconductor	RRID:SCR_012621
clusterProfiler (v4.18.1)	Bioconductor	RRID:SCR_016884
biomaRt (v2.66.0)	Bioconductor	RRID:SCR_019214
org.Hs.e.g.,.db (v3.22.0)	Bioconductor	RRID:SCR_024739
GemomicRanges (v1.62.0)	Bioconductor	N/A
rtracklayer (v1.60.3)	Bioconductor	RRID:SCR_021325
Biostrings (v2.78.0)	Bioconductor	RRID:SCR_016949
ComplexHeatmap (v2.26.0)	Bioconductor	RRID:SCR_017270
circlize (v0.4.16)	CRAN	RRID:SCR_002141
ggplot2 (via tidyverse (v2.0.0))	CRAN	RRID:SCR_014601

**Other**		

5 μm Millex™-SV Filter Unit	Sigma-Aldrich	Cat# SLSV025LS
0.22 μm Syringe Filter	Sarstedt	Cat# 83.1826.001
Amicon® Ultra Centrifugal Filter, 100 kDa MWCO	Sigma-Aldrich	Cat# UFC910008
Multiskan FC Microplate Photometer	Thermo Fisher Scientific	Cat# 51119000
Infinite® M Nano Microplate Reader	Tecan	Cat# 30190086
TC20 Automated Cell Counter	Bio-Rad	RRID:SCR_025462
CFX96 C1000 Real-Time PCR Detection Systems	Bio-Rad	RRID:SCR_018064
Agilent 2100 Bioanalyzer	Agilent	RRID:SCR_018043
Fluorometer Qubit	Thermo Fisher Scientific	RRID:SCR_020553

### Experimental model and study participant details

#### Cell lines

Human A549 lung epithelial cells (male) and HT-29 colorectal epithelial cells (female) were provided by the laboratory of Prof. Varpu Marjomäki (University of Jyväskylä). A549 and HT-29 cells were maintained at 37 °C with 5 % CO_2_ in DMEM supplemented with 10 % fetal bovine serum (FBS), 1 % GlutaMAX, and 1 U/mL penicillin/streptomycin. The cell lines were not independently authenticated in this study. Both cell lines were tested monthly for mycoplasma contamination by real-time PCR using primers Myc_526_F and Myc_1031_R and the p_m16S plasmid as a positive control. Cells between passages 10 and 25 were used for experiments.

#### Bacterial strains

*Pseudomonas aeruginosa* strain PA14 and strain 573, a clinical isolate originally obtained from bone marrow interstitial fluid,^32^^,^^64^^,^^65^ were used in this study. Bacterial strains were routinely cultured in Luria-Bertani Lennox (LB) medium at 37 °C with agitation at 200 rpm. Phage-resistant *P. aeruginosa* PA14 clones generated in this study were stored at −80 °C in LB supplemented with 15 % glycerol and cultured under the same conditions as the parental strain.

#### Bacteriophage

The lytic *P. aeruginosa* bacteriophage DMS3vir^69^ was provided by Prof. Ville Friman (University of Helsinki, Finland) and propagated using *P. aeruginosa* PA14 as the bacterial host. Phage stocks were stored at 4 °C.

### Method details

#### Cell seeding and mucin treatment

For experiments, A549 and HT-29 cells were seeded in 96-well plates at 1 x 10^5^ and 2 x 10^5^ cells per well, respectively. Mucin-enriched conditions were generated using type III porcine gastric mucin (PGM), a mucin source that replicates structural features of human MUC5AC and has been previously used in phage-bacteria-mucin studies.^30^^,^^32^^,^^39^^,^^40^^,^^56^^,^^67^^,^^79^ A 2 % (w/v) PGM stock solution was prepared in PBS, autoclaved, filtered through 5-μm filters, and diluted in culture medium to the concentrations indicated for each experiment. Control groups received an equivalent volume of PBS. Human TNF (100 ng/mL) was used as positive control for qPCR assays, and 20 % DMSO was used as a positive control for cell viability assays.

#### Bacterial preparation and growth assays

For experiments, single colonies were inoculated into 5 mL LB and cultured overnight at 37 °C with agitation at 200 rpm. Cultures were diluted 1:1 with fresh LB and incubated for an additional 1 h under the same conditions. Bacteria were harvested by centrifugation at 4,000 x *g* for 5 min at room temperature (∼22 °C), washed with PBS, and resuspended in LB or antibiotic-free DMEM supplemented with 10 % FBS and 1 % GlutaMAX, as appropriate for each experiment. Bacterial suspensions were standardized to OD_600_ = 0.3 (∼2 x 10^8^ CFU/mL) before further dilution to the required inoculum. Bacterial concentrations were determined by CFU plating or OD_600_ measurements using a Multiskan FC microplate photometer. For growth-curve experiments, OD_600_ was measured every 30 min at 37 °C with shaking using an Infinite M Nano microplate reader.

#### Cell viability assay

Following the indicated experimental treatments, culture supernatants and cells were collected into microcentrifuge tubes. Adherent cells were detached with 0.05 % trypsin for 5 min at 37 °C and pooled with the corresponding supernatant. Samples were centrifuged at 270 x *g* for 5 min at 4 °C, and cell pellets were resuspended in DMEM supplemented with 10 % FBS. Cell suspensions were mixed 1:1 with Trypan Blue, transferred to cell-counting chambers, and viable and non-viable cells were quantified using a TC20 automated cell counter.

#### RNA extraction, reverse transcription, and real-time PCR

Total RNA was extracted using the RNeasy Mini Kit. Reverse transcription was performed using the High-Capacity cDNA Reverse Transcription Kit with 200 ng total RNA as input. Quantitative real-time PCR (qPCR) was performed using GoTaq qPCR Master Mix on a CFX96 C1000 Real-Time PCR Detection System. Reactions contained 2 μL of 1:10-diluted cDNA and primers at a final concentration of 0.2 μM in a total reaction volume of 10 μL. Human *18S* rRNA was used as the reference gene for analysis of human gene expression. *P. aeruginosa oprL* was used as bacterial reference gene. Relative gene expression was calculated using the 2^-ΔΔCt^ method in CFX Maestro v2.3 (Bio-Rad). Primer sequences are provided in the key resources table.

#### Bacterial internalization assay

Bacterial internalization was quantified using a gentamicin protection assay. Following bacterial exposure, epithelial cells were incubated with 400 μg/mL gentamicin for 1 h at 37 °C to eliminate extracellular bacteria. The effectiveness of gentamicin treatment is shown in Figure S5. Following treatment, cells were washed with PBS, and intracellular bacteria were released by treatment with 0.05 % trypsin and 0.2 % Triton X-100. Released bacteria were quantified by serial dilution and CFU plating. Control groups without epithelial cells were included as negative controls.

#### Bacterial adhesion assay

To quantify bacterial adhesion to epithelial cells, culture supernatants were removed and cells were gently washed three times with PBS to remove non-adherent bacteria. Epithelial cells were subsequently lysed to recover total cell-associated bacteria, comprising both adherent and intracellular bacteria. In parallel, intracellular bacteria were quantified using the gentamicin protection assay described above. The number of adherent bacteria was calculated by subtracting the number of intracellular bacteria from the total number of cell-associated bacteria.

#### Bacterial secretome cytotoxicity assay

*P. aeruginosa* cultures were incubated in DMEM supplemented with 10 % FBS under static conditions at 37 °C for 6 or 24 h. Control medium without bacteria was incubated under identical conditions. Following incubation, bacterial cultures were centrifuged at 4,000 x *g* for 5 min, and the resulting supernatants were passed through sterile 0.22-μm syringe filters to remove viable bacteria while retaining soluble secreted components. A549 cells were exposed to the resulting bacteria-free secretomes for 5 h at 37 °C and 5 % CO_2_. Epithelial cytotoxicity was subsequently quantified using the cell viability assay described above. CFU plating was performed to confirm the absence of viable bacteria from the filtered secretomes.

#### Pyocyanin production

Pyocyanin production was quantified by chloroform-HCl extraction. Overnight bacterial cultures were diluted to 2 x 10^8^ CFU/well in LB and incubated at 37 °C for 6 or 48 h in 12-well plates using a DTS-4 shaker (ELMI). Cultures were centrifuged at 5,000 x *g* for 10 min, and supernatants were mixed with chloroform at a 5:3 ratio by repeated vortexing. The resulting organic phase was mixed with 0.2 M HCl at a 2:1 ratio and vortexed. Absorbance of the aqueous phase was measured at 520 nm using an Infinite M Nano microplate reader.

#### Biofilm formation assay

Overnight bacterial cultures were diluted to OD_600_ = 0.1 and seeded into 96-well plates. Plates were incubated under static conditions at 37 °C for 24 h. Following incubation, plates were washed with water, and biofilms were stained with 0.1 % crystal violet for 20 min at room temperature. Excess stains were removed by washing, and the plates were allowed to dry. Biofilm-associated crystal violet was subsequently solubilized using 96 % ethanol, and absorbance was measured at 595 nm using a Multiskan FC microplate photometer.

#### Bacteriophage propagation, purification, and titration

DMS3vir was propagated using *P. aeruginosa* PA14 as the bacterial host. Phage lysates were filtered through 0.22-μm filters, treated with 10 % chloroform, and concentrated using 100-kDa molecular-weight-cutoff Amicon centrifugal filters. Phage preparations were subsequently purified using the EndoTrap HD 1/1 Kit. Endotoxin concentrations were measured using the Pierce Chromogenic Endotoxin Quant Kit. Purification reduced endotoxin levels from 7,158 EU/10^9^ PFU to 2 EU/10^9^ PFU. Phage titers were determined using the double-layer agar assay, and purified phage stocks were stored at 4°C.

#### Isolation of phage-resistant bacteria

Phage-resistant *P. aeruginosa* clones were isolated from A549 cultures following incubation with DMS3vir for 24 h. Three independent colonies were obtained from each of two phage-exposed conditions: BIM, corresponding to A549 cells with DMS3vir, and BIM(M), corresponding to A549 cells with DMS3vir and 0.2 % mucin. Control isolates comprised PA14(WT), the parental strain; PA14(C), recovered following exposure to A549 cells; and PA14(M), recovered following exposure to A549 cells in the presence of 0.2 % mucin. Isolated colonies were re-streaked at least three times to obtain purified clones and stored at −80 °C in 15 % glycerol.

#### Bacterial motility assays

For swarming motility assays, bacterial cultures were diluted to OD_600_ = 0.1, and 2 μL of each suspension was spotted onto LB soft agar. Plates were incubated under static conditions at 37 °C for 24 h. Swarming patterns were imaged alongside a ruler, and colony diameters were quantified using ImageJ2 v2. For twitching motility assays, bacterial colonies were stabbed through the agar to the bottom of LB agar plates using inoculation needles and incubated at 37 °C for 48 h. Following incubation, the agar was removed, and the plates were stained with 0.1 % crystal violet. Twitching zones were imaged and their diameters quantified using ImageJ2 v2.

#### RNA sequencing and analysis

Total RNA was extracted from A549 cells from control and mucin-treated groups (*n* = 3 biological replicates per group) using the RNeasy Mini Kit. RNA integrity was assessed using an Agilent 2100 Bioanalyzer, and samples with RNA integrity number (RIN) >8 were used for sequencing. Strand-specific mRNA libraries were commercially prepared and sequenced by BGI Tech (China) using the DNBseq platform with 150-bp paired-end reads, generating approximately 20–30 million reads per sample. Sequencing data were processed using the Chipster RNA-seq workflow (accessed October 2025) using default parameters. Differential expression was independently evaluated using DESeq2 and edgeR. Genes with a false discovery rate (FDR) <0.05 in both analyses were retained as the intersection of the DESeq2 and edgeR results. Gene annotations were obtained using biomaRt and merged with org.Hs.eg.db. Principal component analysis was performed using the 1,000 most variable genes. Data visualization was performed using ggplot2 and ComplexHeatmap. Gene Ontology enrichment was evaluated by over-representation analysis (ORA) and complemented by gene set enrichment analysis (GSEA) using clusterProfiler. Enriched terms were curated and visualized as bar plots. Additional description available in Data availability.

#### Whole-genome sequencing and analysis

Genomic DNA was extracted from *P. aeruginosa* isolates using the DNeasy Blood & Tissue Kit and quantified using a Qubit fluorometer. Sequencing libraries with insert sizes ≤800 bp were prepared and sequenced by BGI Tech using the DNBseq platform with 150-bp paired-end reads. Sequencing of the BIM2 sample failed because of technical issues, and this sample was therefore excluded from downstream whole-genome sequencing analyses. Sequencing reads were filtered using fastp and quality-control metrics were summarized using MultiQC. Filtered reads were aligned to *P. aeruginosa* UCBPP-PA14 reference genome (NC_008463.1) using BWA-MEM. Alignments were processed using SAMtools and GATK MarkDuplicates. Variants were called using SAMtools mpileup and VarScan. Variants were annotated in R using VariantAnnotation together with the *P. aeruginosa* UCBPP-PA14 GFF annotation and UniProt information. Synonymous background variants were excluded from downstream analyses. Heatmaps were generated using ComplexHeatmap, and genes carrying variants were grouped according to KEGG pathway/function and visualized using stacked bar plots. *De novo* genome assemblies were generated using SPAdes. CRISPR arrays were detected using MinCED, parsed in R, and visualized as tile plots. Additional description available in Data availability.

### Quantification and statistical analysis

Statistical analyses were performed using GraphPad Prism v10.3.0 and R v4.5.1. Data are presented as mean ± SEM, unless otherwise indicated. The exact number of independent biological replicates (n) for each experiment, the definition of n, and the statistical test applied are reported in the corresponding figure legends. Exact *p* values are reported in the figures or corresponding figure legends, and *p* < 0.05 was considered statistically significant. Comparisons between two groups were performed using two-tailed *Student's t-tests*. Comparisons involving more than two groups were performed using one-way ANOVA followed by *Tukey's* multiple-comparisons test. Experiments involving two independent variables were analyzed using two-way ANOVA followed by *Sidak's* multiple-comparisons test. For RNA-seq analysis, three independent biological replicates were analyzed per experimental group. Differential gene expression was independently assessed using DESeq2 and edgeR. DESeq2 statistical significance was determined using *Wald* tests, whereas edgeR used *quasi-likelihood F-tests*. *p* values were adjusted for multiple comparisons using the *Benjamini-Hochberg* method, and genes with a false discovery rate (FDR) < 0.05 in both analyses were considered differentially expressed. The RNA-seq sample size was based on detecting fold changes >1.5 with 80 % power at α = 0.05. No inferential statistical tests were applied to CRISPR spacer-acquisition data, which are presented descriptively. No experimental data were excluded from analysis unless otherwise specified. Whole-genome sequencing of BIM2 was excluded because sequencing failed due to technical issues.

## Supplemental information

Document S1. Figures S1–S5

Data S1. Dataset of differentially expressed gene analysis of A549 cells exposed to mucin, related to Figures 1 and S1

Data S2. Dataset of GO enrichment analysis of A549 cells exposed to mucin, related to Figure 1

Data S3. Dataset of whole genome sequencing analysis of *P. aeruginosa* PA14 isolates, related to Figure S4

Data S4. Dataset of functional/pathway annotation analysis of mutated genes in *P. aeruginosa* PA14 isolates, related to Figure S4

Data S5. Dataset of CRISPR spacer acquisition analysis of *P. aeruginosa* PA14 isolates, related to Figure S4
