# Supplementary material for: Mucin-rich environment shapes Pseudomonas aeruginosa cytotoxicity and phage-mediated epithelial protection
Source: iScience. 2026 Oct 28;29(11):118016. doi: 10.1016/j.isci.2026.118016 (PMC13733329; doi:10.1016/j.isci.2026.118016)
Supplement: Supplementary file 1 [file mmc1.pdf]

iScience, Volume *29*

Supplemental information

Mucin-rich environment shapes *Pseudomonas aeruginosa* cytotoxicity and phage-mediated epithelial protection

Daniel O. Patricio, Katri Niitti, Iina Salminen, Lucilene W. Granella, Valtteri Suokko, Gabriel M.F. Almeida, and Lotta-Riina Sundberg

Supplemental figure 1

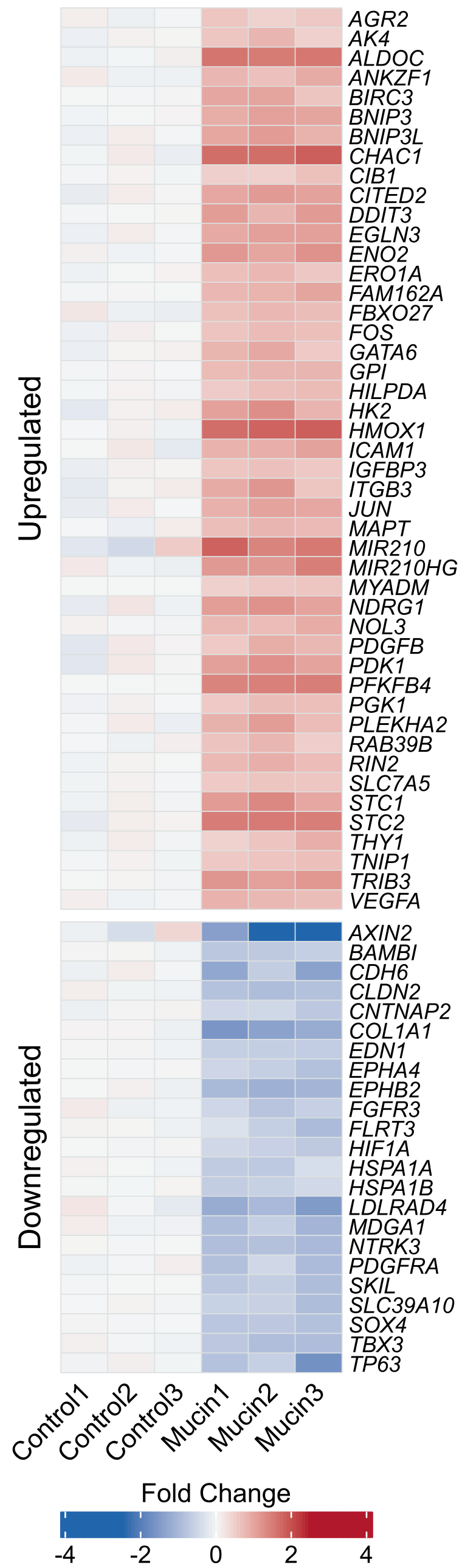

Figure S1. Transcriptomic analysis of A549 cells exposed to mucin-rich environment. A549 cells were incubated with 0.2 % mucin or control medium for 18 h. Heatmap of significantly differentially expressed genes between conditions (FDR < 0.05, log2 fold change > 1) from GO biologic process terms. Genes are hierarchically clustered and grouped into upregulated (top) and downregulated (bottom) sets. Columns represent biological replicates. Color scale indicates expression values.

Supplemental figure 2

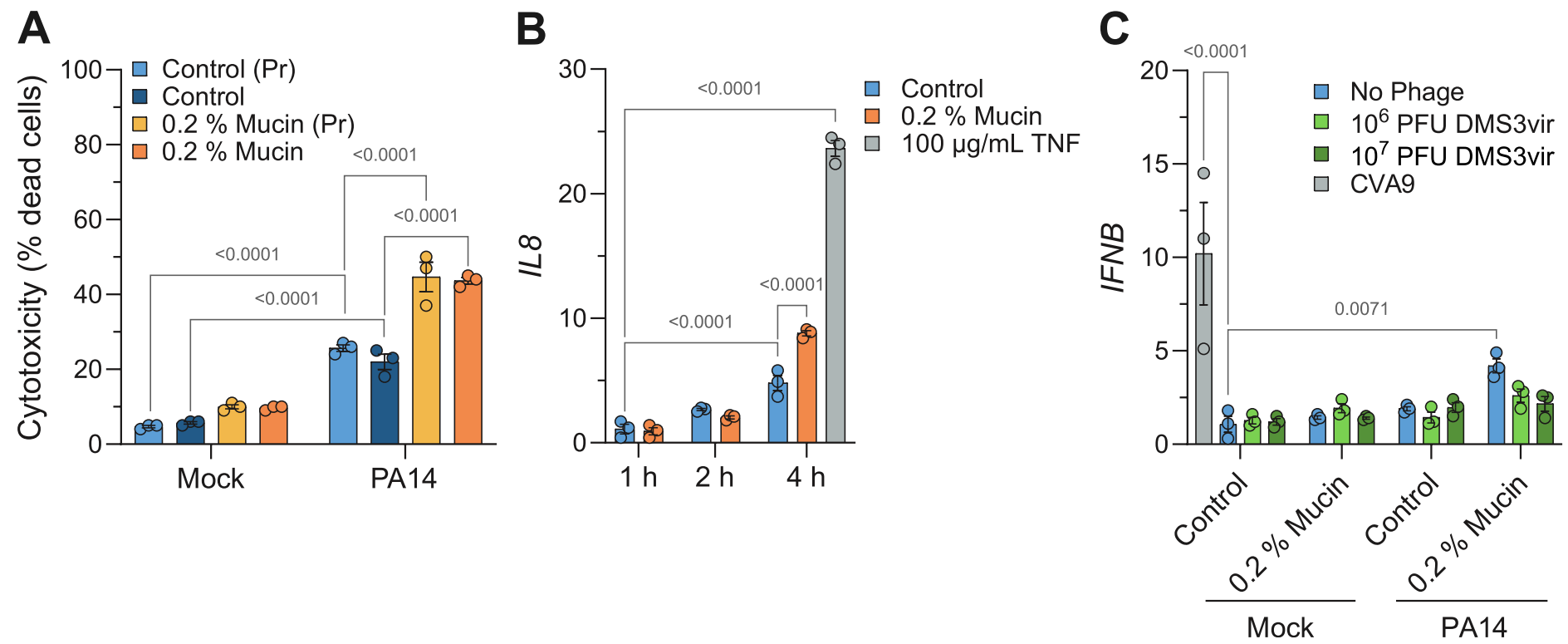

Figure S2. *P. aeruginosa* cytotoxicity and transcriptional expression of *IL8* and *IFNB* in infected epithelial cells. (**A**) Bacterial cytotoxicity on A549 cells pre-exposed (Pr) to mucin or co-exposed to mucin and PA14 for 4 h. (**B**) Time-course analysis of relative *IL8* mRNA expression in A549 cells pre-exposed to mucin for 18 h prior *P. aeruginosa* PA14 infection (1 x 10^5 CFU) for 4 h. The 100 µg/mL TNF incubation for 30 min was used as a positive control. (**C**) Relative expression of *IFNB* mRNA of A549 cells pre-incubated with DMS3vir and mucin for 18 h, followed by infection with *P. aeruginosa* PA14 (1 x 10^5 CFU) for 4 h. *Coxsackievirus* A9 (CVA9) infection (MOI 0.001) was used as a positive control. Quantitative data represent mean ± SEM from at least two independent experiments. Statistical comparisons were performed using two-way ANOVA with multiple comparisons and Sidak's post hoc test; *p*-values and replicate numbers are indicated.

Supplemental figure 3

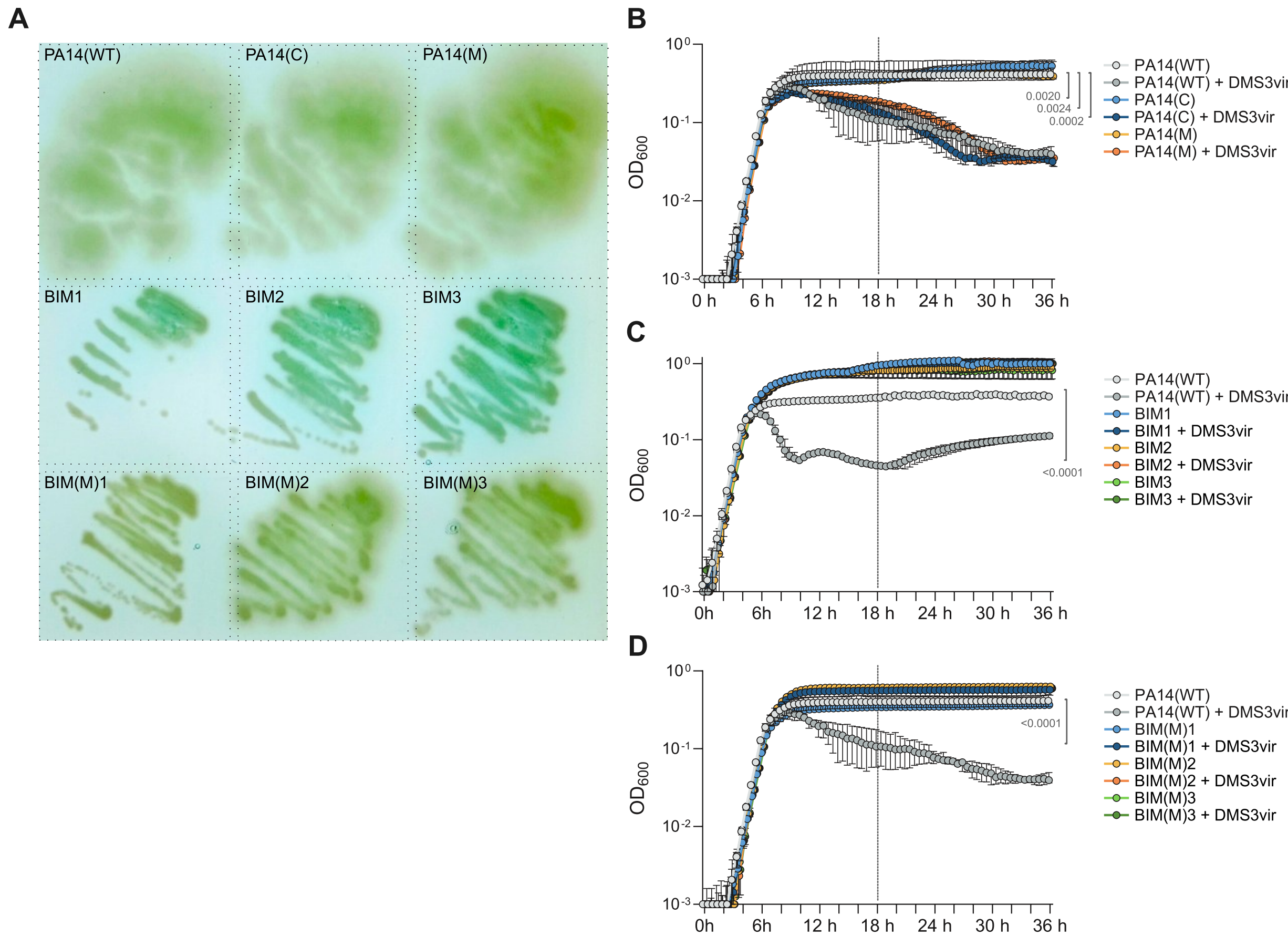

Figure S3. Phage-resistance analysis of bacteriophage-insensitive mutants (BIM) *P. aeruginosa* strains. (**A**) Representative colony images of *P. aeruginosa* PA14 clones isolated after 24 h co-culture at 1 x 10^5 CFU with A549 cells pre-exposed to 0.2 % mucin and DMS3vir phage (1 x 10^7 PFU). (**B**) Bacterial growth curves of PA14 control clones, (**C**) BIM, and (**D**) BIM(M) exposed to DMS3vir (1 x 10^7 PFU). Growth curves represent four replicates. BIM and BIM(M) refer to phage-resistant clones isolated in the absence or presence of 0.2 % mucin, respectively.

Supplemental figure 4

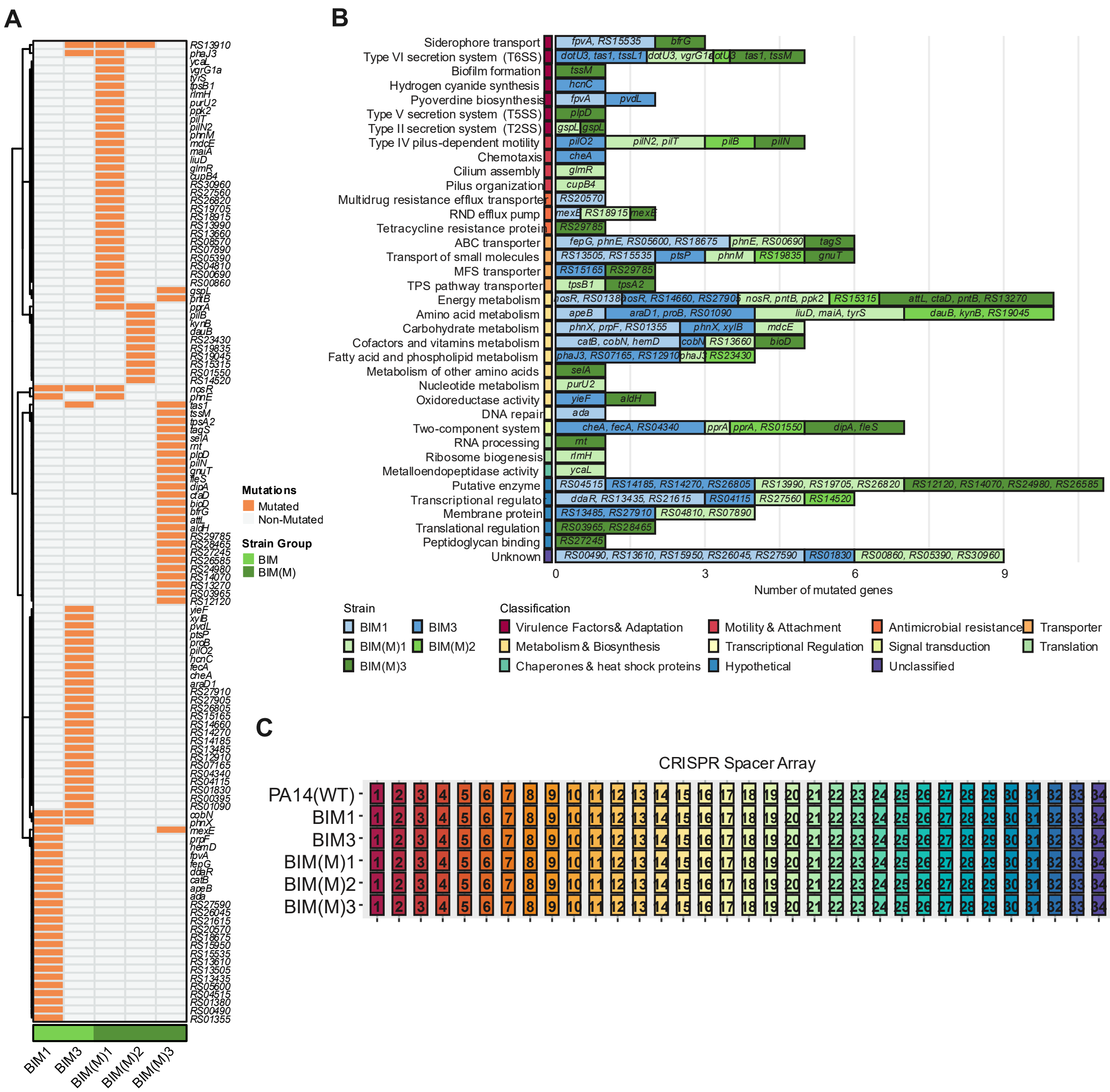

Figure S4. Whole genome sequencing analysis of bacteriophage-insensitive mutants (BIM) ***P. aeruginosa*** **strains.** (**A**) Heatmap of mutated genes BIM and BIM(M) clones. Columns represent biological replicates. BIM and BIM(M) refer to phage-resistant clones isolated in the absence or presence of 0.2 % mucin, respectively, as described in Figure 7. Mutated genes shared with PA14(WT), PA14(C), or PA14(M) were excluded. (**B**) Functional/pathway annotation of whole genome sequenced genes mutated in BIM1-2 and BIM(M)1-3 strains. Mutated genes shared with PA14(WT), PA14(C), or PA14(M) were excluded. (**C**) CRISPR spacer acquisition analysis.

Supplemental figure 5

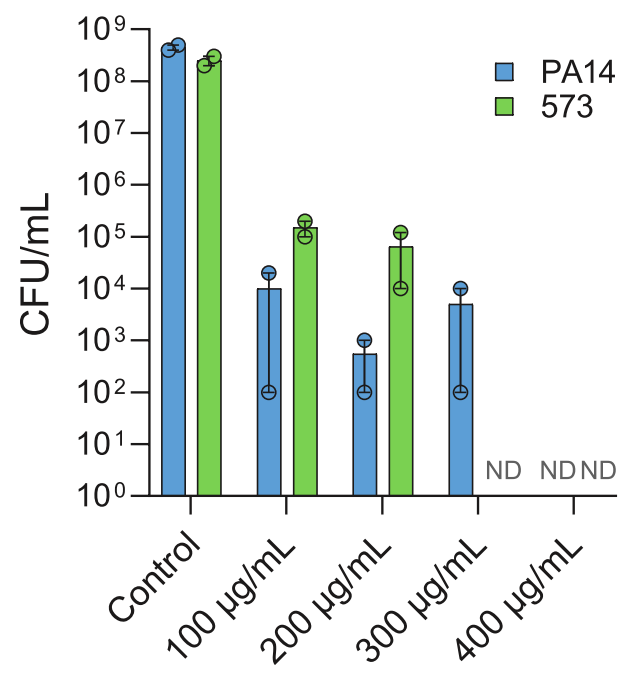

Figure S5. Antibacterial effects of gentamicin on *P. aeruginosa*. *P. aeruginosa* strains PA14 and 573 exposed to gentamicin for 4 h. Antimicrobial effects of gentamicin were analyzed by bacterial colony-forming unit (CFU) quantification. ND: Non-detected. Statistical comparisons were performed using two-way ANOVA with multiple comparisons and Sidak's post hoc test; replicate numbers are indicated.
